# ThalPred: a web-based prediction tool for discriminating thalassemia trait and iron deficiency anemia

**DOI:** 10.1186/s12911-019-0929-2

**Published:** 2019-11-07

**Authors:** V. Laengsri, W. Shoombuatong, W. Adirojananon, C. Nantasenamart, V. Prachayasittikul, P. Nuchnoi

**Affiliations:** 1 Center for Research and Innovation, Faculty of Medical Technology, Mahidol University, Bangkok, Thailand; 2 Department of Clinical Microscopy, Faculty of Medical Technology, Mahidol University, Bangkok, Thailand; 3 Center of Data Mining and Medical Informatics, Faculty of Medical Technology, Mahidol University, Bangkok, Thailand; 4 Department of Clinical Microbiology and Applied Technology, Faculty of Medical Technology, Mahidol University, Bangkok, Thailand

**Keywords:** Thalassemia trait, Iron deficiency anemia, Machine learning, Support vector machine, Random forest, Discrimination, Decision making

## Abstract

**Background:**

The hypochromic microcytic anemia (HMA) commonly found in Thailand are iron deficiency anemia (IDA) and thalassemia trait (TT). Accurate discrimination between IDA and TT is an important issue and better methods are urgently needed. Although considerable RBC formulas and indices with various optimal cut-off values have been developed, distinguishing between IDA and TT is still a challenging problem due to the diversity of various anemic populations. To address this problem, it is desirable to develop an improved and automated prediction model for discriminating IDA from TT.

**Methods:**

We retrospectively collected laboratory data of HMA found in Thai adults. Five machine learnings, including *k*-nearest neighbor (*k*-NN), decision tree, random forest (RF), artificial neural network (ANN) and support vector machine (SVM), were applied to construct a discriminant model. Performance was assessed and compared with thirteen existing discriminant formulas and indices.

**Results:**

The data of 186 patients (146 patients with TT and 40 with IDA) were enrolled. The interpretable rules derived from the RF model were proposed to demonstrate the combination of RBC indices for discriminating IDA from TT. A web-based tool ‘ThalPred’ was implemented using an SVM model based on seven RBC parameters. ThalPred achieved prediction results with an external accuracy, MCC and AUC of 95.59, 0.87 and 0.98, respectively.

**Conclusion:**

ThalPred and an interpretable rule were provided for distinguishing IDA from TT. For the convenience of health care team experimental scientists, a web-based tool has been established at http://codes.bio/thalpred/ by which users can easily get their desired screening test result without the need to go through the underlying mathematical and computational details.

## Background

Anemia is the condition of decreased number of red blood cells (RBCs) or of the concentration of hemoglobin (Hb). Anemia is a health problem affecting both developing and developed countries. The global prevalence of anemia in 2010 was 32.9% and it was especially common in Central Africa, the Middle East, the Mediterranean and Southeast Asia [1]. It can occur from one of three causes, acute blood loss, increased hemolysis or ineffective hematopoiesis.

In Thailand, both iron deficiency anemia (IDA) and thalassemia trait (TT) are highly prevalent. Iron is an important element in our body, being a component of many enzymes and playing a role in hemoglobin synthesis. Therefore, a lack of iron can lead to IDA. The prevalence of IDA among Thai people is estimated to be 1.5–8% [2]. Thalassemia is an inherited hematological disorder that is caused by abnormal production of alpha (α)- or beta (β)- globin chains. The prevalence of TT is approximately 20–25% in the Thai population [3]. The levels of serum ferritin, serum iron, total iron binding capacity and percentage of transferrin saturation are the most commonly used assays to confirm IDA [4]. Meanwhile, Hb testing via high performance liquid chromatography or capillary electrophoresis to detect abnormal Hb levels or DNA analysis are the assays commonly used for TT diagnosis [5–7]. In practice, these latter techniques are not available in routine laboratories as they require special machines, and are time- and cost-consuming. Basically, when patients were assumed to be TT or IDA, clinician often prescribed the cassette of hematological tests covering both TT and IDA diagnosis. These laboratory work-ups consume personnel work load and governmental budget. This leads to financial crisis for national health care of low-middle income countries. In order to guide clinician for rational lab use, we therefore developed web-based tool for assisting clinician to prescribe rational and cost-effective laboratory testing for TT and IDA diagnosis. Multiple formulas and indices have been proposed for such discrimination including the Bessman index (BI) [8], Ehsani formula (EF) [9], England & Fraser index (E&F) [10], Green & King index (G&K) [11], Mentzler index (MI) [12], Red Cell Distribution Width index (RDWI) [13], Ricerca index (RI) [14], Shine and Lal index (S&L) [15], Siridah index (SI) [16], Srivastava formula (SF) [17], Sirachainan formula (SiF) [18], Kandhro 1 formula (KF1) [19] and Kandhro 2 formula (KF2) [19].

The aforementioned discriminant indices and formulas yielded quite encouraging prediction results. However, the prediction results across different populations, especially in sensitivity and specificity, are still unsatisfactory. Some of studies are contentious due to difference of gender, age or ethnicity [20–22]. In order to improve prediction results, the computation of optimal cut-off values for specific populations in different countries is needed [23, 24]. The potential of machine learning techniques has been demonstrated for near-term translational impact. For instance, in the case of the biomedicine, major applications of machine learning are medical/radiological diagnosis and drug discovery. Thus, it may be possible to develop discriminant models based on machine learning techniques for providing effective large-scale analyses of laboratory data. To the best of our knowledge, only one discriminant model has been proposed to differentiate IDA and TT in the Thai population [18]. In this study, we exerted an effort to develop a powerful model to discriminate IDA from TT using a support vector machine (SVM) which we named *ThalPred*.

The previous works had demonstrated the meaningful of data mining and the increasing of computational power in various aspect of biomedical application [25–28]. The present study aims to establish a reliable and interpretable computational model. Therefore, the important procedures as the following are considered: (i) collect clear and reliable laboratory dataset for training and validating; (ii) demonstrate characteristic or descriptor of dataset for intrinsic properties prediction; (iii) identify feature of importance for improving interpretability; (iv) develop a simple and interpretable model; (v) perform rigorous cross validation for analyzing internal and external predictive power; (vi) develop affordable and user friendly based web tool for implementation in the healthcare community.

## Material and methods

### Data collection

This was a retrospective study of encoded and unlinked clinical laboratory data obtained from the Center of Medical Laboratory Services, the Faculty of Medical Technology, Mahidol University from the period July 2014 to September 2016. The abbreviations of hematological laboratory testing were listed in Table 1. We collected 237 sets of hematological data from Thai subjects, age 18 to 60 years, that showed hypochromic microcytic anemia (HMA) and were diagnosed as reflecting IDA or TT by two independent medical technologists. Other causes of HMA, such as hypothyroidism, anemia of chronic disorders, hepatitis B / C / or D infection, or *Helicobacter pylori* infection, were excluded from the study in order to specify IDA and TT only. Hemoglobin electrophoresis and serum ferritin results were analyzed to discriminate TT and IDA, respectively. The level of ferritin was determined by electrochemiluminescence method (Cobas® 2014© Roche, Switzerland). Variant hemoglobin testing was performed using low pressure liquid chromatography (Bio-Rad Laboratories, USA). The final dataset consisted of 186 subjects (146 TT and 40 IDA cases) which were used for internal and external analyses. The study was conducted under the approval of the Mahidol University Central Institutional Review Board (MU-CIRB; CODE No. MU-CIRB 2016/084.0311). We received a participant consent waiver from MU-CIRB. All information of subjects was de-identified prior data analysis.

**Table 1:** List of laboratory testing abbreviation used in this study

Full Name	Abbreviation
Hemoglobin	Hb
Hematocrit	Hct
Hypochromic microcytic anemia	HMA
Iron deficiency anemia	IDA
Mean corpuscular volume	MCV
Mean corpuscular hemoglobin	MCH
Mean corpuscular hemoglobin concentration	MCHC
Red blood cell	RBC
Red blood cell distribution width	RDW
Thalassemia trait	TT

### Statistical analyses

The statistical analysis of this study was computed using SPSS software, version 20 (SPSS Inc., New York, Armonk, USA). As an exploratory statistical analysis, univariate statistical analysis using mean and standard deviation (SD) was performed to investigate the different patterns and trends of individual hematological parameters between the data of groups TT and IDA. The Kolmogorov-Smirnov test was used for normality testing. Since the data of this study was not normally distributed. The comparison between groups TT and IDA was performed by using the Mann-Whitney U test. A *p*-value < 0.05 was considered significant. Furthermore, in order to perform multivariate statistical analyses amongst RBC indices, principal component analysis (PCA) was performed. PCA has probably been the most popular technique to perform multivariate statistical analysis for the tasks of data exploration and pattern recognition. The advantages of PCA are to: (i) extract the most important information and represent it with only a few dimensions, called principal components (PCs); (ii) compress the dimensions of the dataset by keeping only the important information; and (iii) analyze the characteristics and structure of the data set. In this study, PCA was performed by using the *FactoMineR* package [29] in R program to represent the distributions of IDA and TT cases, and identify the RBC indices important for distinguishing IDA and TT cases.

### Models construction

**Fig. 1:**
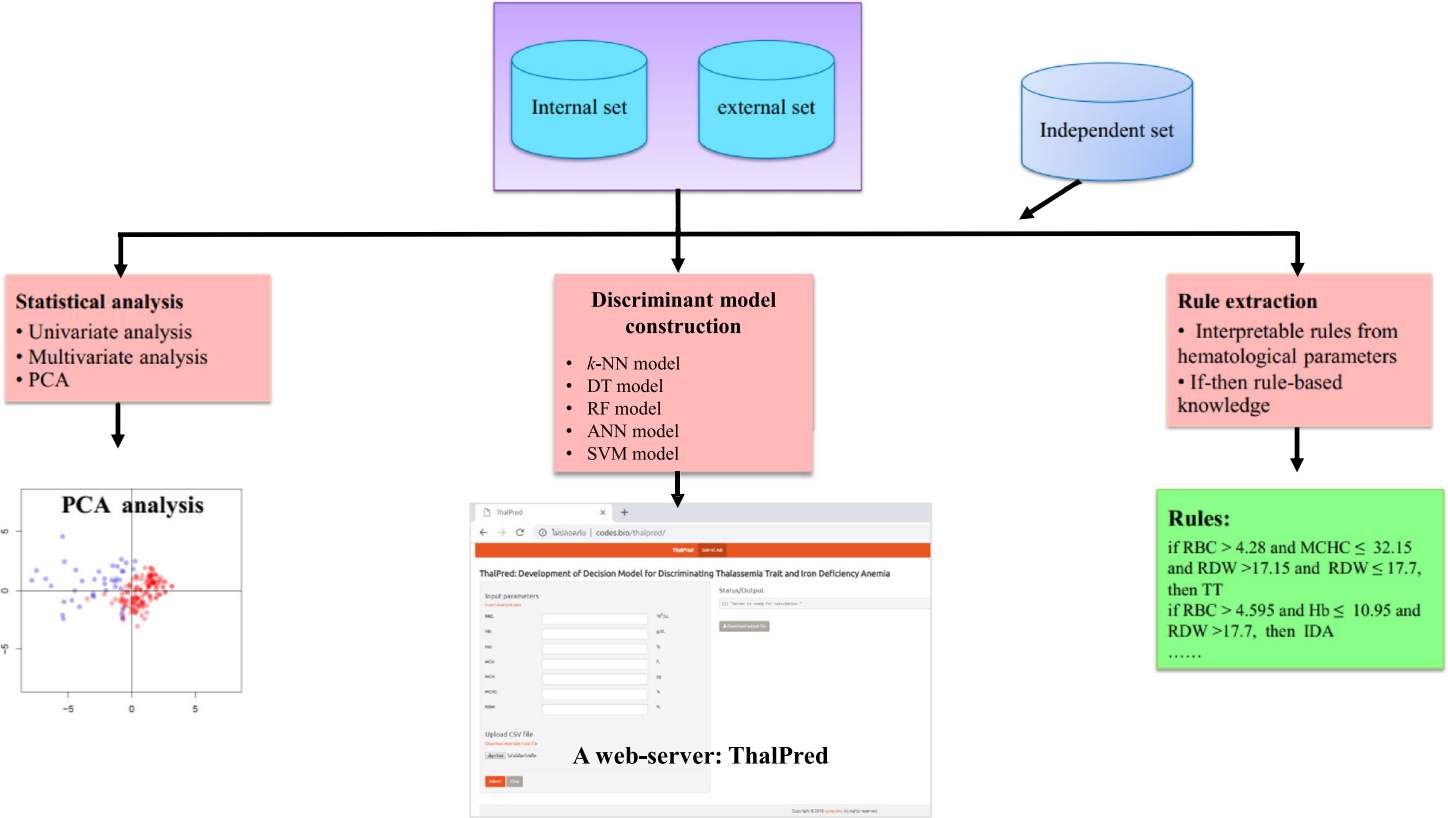
The workflow of the computation model of ThalPred for discriminating IDA from TT and providing the set of interpretable rules

The following five popular computational models, namely *k*-nearest neighbor (*k*-NN), decision tree (DT), random forest (RF), artificial neural network (ANN) and support vector machine learning (SVM), were applied to develop discriminant models for effectively distinguishing TT from IDA. The fundamental and associated parameter optimization for the five classifiers are briefly described (Fig. 1).

*k*-NN is one of the most popular and lazy machine learning algorithms for a variety of problem domains [25, 30, 31]. This algorithm is conceptually based on a distance function, such as the Euclidean distance, to measure the similarity between a pair of training and unknown datasets. For obtaining the best *k*-NN model, the suitable number of neighbors (*k* ∈ {5, 7, 9,..., 43} was determined by using the *caret* package [28, 32] over 5-fold cross-validation (5-fold CV) scheme.

DT model can be used in the classification and regression tasks by constructing a model in the form of a tree structure. Herein, we constructed the DT model by implementing the J48 algorithm from the RWeka package in R program (version 3.3.2) [33] using default parameters. Briefly, the J48 algorithm is a re-implementation of the C4.5 algorithm [34] based on Javascript. The feature with the highest information gain is select to build a model. Finally, because of its built-in feature selector, the DT model will provide the feature usage score for ranking the feature importance.

RF is an ensemble classification and regression tree (CART) classifier [34, 35]. The RF model is a classifier derived from gathering many weak CART trees for improving the prediction performance. To construct the model, RF takes advantage of two well-known machine learning algorithms, i.e. bagging and random feature selection. To estimate informative features, RF model utilizes two measures, i.e. mean decrease in accuracy and Gini index [35]. In this study, the RF classifier was implemented using the *randomForest R* package [34]. To obtain an optimal RF model, two parameters, namely, *ntree* (i.e., the number of trees used for constructing the RF classifier) and *mtry* (i.e., the number of random candidate features), were tuned by using a grid search procedure based on 5-fold cross-validation (5-fold CV), where *ntree* ∈ {100, 200, 300, 400, 500} was determined, while *mtry* was estimated using the tuneRF function in the *randomForest* R package [34].

ANN is computing systems originally inspired by the way biological nervous systems process information [36]. Previously, many researchers reported that ANN accomplished well in many domains, such as protein sequence analysis, image recognition, speech recognition, and natural language processing [27, 37, 38]. In practice, there are two important types of ANN, i.e. the perceptron and the sigmoid neuron, while stochastic gradient descent is known as the standard estimating parameter algorithm for ANN. For achieving the best ANN model, two parameters, namely, *size* (i.e., the number of hidden nodes) and *decay* (i.e., the number of weight decay), were subjected to optimization. Particularly, *size* ∈ {1, 2, 3,..., 10} and *decay* ∈ {0.1, 0.2, 0.3, 0.4, 0.5} were determined by using the *caret* package [28, 32] over a 5-fold CV scheme.

SVM is a statistical learning approach based on the principle of structure-risk minimization and a kernel method (as proposed by Vapnik [39]) which are used to construct a maximum-margin-separating hyperplane for distinguishing the two classes of interest. The radial basis function kernel was used to transform the original feature space into a higher dimensional space in which the SVM classifier can linearly separate the inherent classes of the dependent variable via a maximum separating hyperplane [40]. Optimization of the SVM parameters consisting of the cost ∈ {2 ^8^, 2 ^7^, 2 ^6^,..., 2^7^, 2^8^} and γ ∈ {2 ^8^, 2 ^7^, 2 ^6^,..., 2^7^, 2^8^} were determined via a grid search spanning the search space evaluated by 5-fold CV scheme using the *e1071*R package [41].

### Cross-validation for identification of discriminant capability

The validation of an empirical predictive model is essential. In order to train and evaluate the discriminant models, the data set of this study was randomly partitioned into internal and external sets with 80 and 20%, respectively, of the data set from both TT and IDA. The internal set was evaluated using the 5-fold CV scheme [42] to confirm the reliability and robustness of the proposed discriminative model. The external set was used to assess the generalizability of the model when extrapolating to unknown samples. To avoid the possibility of bias arising from a single data split upon model training, data splitting was performed for 100 independent iterations. The final prediction performances of the 5-fold CV and external validation tests of the proposed discriminative model were reported by using the mean and standard deviation values of analyzed parameters (Fig. 1).

### Rule extraction

In addition to model accuracy and ability to discriminate IDA from TT, there is a possibility that a simple and meaningful rule may be extracted from an RF model. From the *nroot* to a leaf node is a rule for a tree. The main purpose of extraction rules is to ease utilization and make the model more interpretable compared to black-box approaches [25] such as SVM. In this study, the extraction rules were obtained by using the R package *inTrees* (interpretable trees) [34] and only 100 decision trees were established for each encoding to train the RF model. *InTrees* is a powerful package for extracting, measuring, pruning, selecting and summarizing from an RF model (Fig. 1).

### Performance assessment

Performance assessment is important step for developing reliable and useful predictor. In order to discriminate TT from IDA, pattern recognition is applied for classification concept. Five standard statistical parameters, namely, accuracy (Ac), sensitivity (Sn), specificity (Sp), Matthew’s correlation coefficient (MCC), and Youden’s index (YI), were addressed to evaluate the predictive performance of the proposed methods. These five parameters were computed as follows [43–46]:

$$ \mathbf{Ac}=\frac{TP+ TN}{\left( TP+ TN+ FP+ FN\right)}\times 100 $$

$$ \mathbf{Sn}=\frac{TP}{\left( TP+ FN\right)}\times 100 $$

$$ \mathbf{Sp}=\frac{TN}{\left( TN+ FP\right)}\times 100 $$

$$ \mathbf{MCC}=\frac{(TP)(TN)-(FP)(Fn)}{\sqrt{\left( TP+ FP\right)\left( TP+ FN\right)\left( TN+ FP\right)\left( TN+ FN\right)}} $$

$$ \mathbf{YI}=\left[\left(\mathrm{Sn}+\mathrm{Sp}\right)-100\right]/100 $$

where TP, FP, TN and FN are true positive, false positive, true negative and false negative, respectively. Furthermore, the receiver operating characteristic (ROC) curve, which plots the true positive rate (Sn) against the false positive rate (1-Sp) at different thresholds, was also used for model evaluation. A predictor with perfect classification has a ROC curve passing through the top-left corner, i.e. Sn = 100% and Sp = 100%. To plot the ROC curve and calculate the value of area under the curve (AUC), the ROCR package [47] was used.

## Results

### Differences in hematological data of TT and IDA using univariate and multivariate analyses

**Fig. 2:**
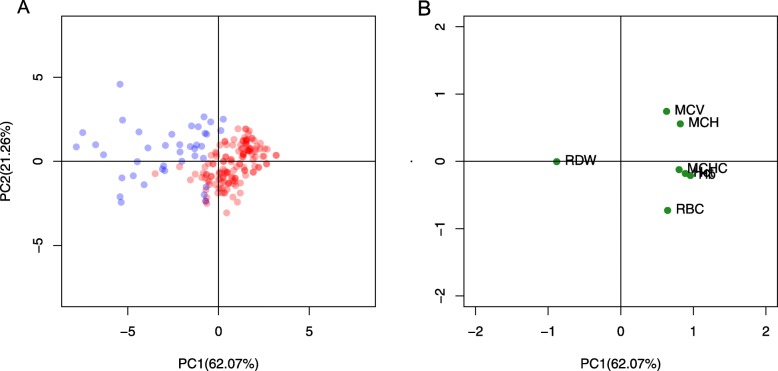
Multivariate analysis using principal component analysis (PCA) of our laboratory data consisting of 146 TT cases (red circles) and 40 IDA cases (blue circles) derived from PCA scores (**a**) and loadings (**b**) plots

HMA data was classified into two groups: 146 TT cases and 40 IDA cases. The characteristics and hematological data of both groups of IDA and TT are summarized in Table 2. Age of two groups was not significantly different (*p-*value 0.435). All RBC parameters were significantly different between the two groups (*p* < 0.05). All averages of parameters in TT group were higher than IDA group except RDW. Furthermore, PCA was applied as multivariate analysis was presented as scores (Fig. 2a) and loadings plots (Fig. 2b). The percentage of variance explained by the first two PCs (83. 33%) were considered sufficient in describing the behavior of the data. The graphs of Fig. 2a and Fig. 2b also revealed that RDW contributes greatly to IDA, while the remaining RBC parameters contribute to TT. These results were well reflected by the mean values of IDA (15.88 ± 1.13) and TT (20. 48 ± 3.23). Interestingly, the outputs of two analyses were consistent. Therefore, these suggested that all RBC indices may be important to discriminate between patients with IDA and those with TT.

**Table 2:** The age and red blood cell parameters of study subjects with thalassemia trait or iron deficiency anemia

Parameters	TT (*N* = 146)	IDA (*N* = 40)	*p*-value
Age (yrs.)	37.79 ± 7.86 (18.00–50.00)	39.15 ± 9.61 (23.00–58.00)	0.435
RBC (10^6^/μL)	5.32 ± 0.48 (4.35–6.83)	4.03 ± 0.96 (1.69–5.77)	< 0.001^*^
Hb (g/dL)	11.99 ± 1.11 (8.10–14.60)	7.97 ± 2.26 (2.50–10.90)	< 0.001^*^
Hct (%)	36.46 ± 4.10 (34.10–42.40)	26.19 ± 6.63 (9.90–34.30)	< 0.001^*^
MCV (fL)	69.49 ± 6.14 (52.30–79.70)	65.12 ± 9.32 (48.70–81.00)	0.010^*^
MCH (pg)	22.71 ± 2.25 (17.70–26.60)	19.65 ± 3.33 (12.50–25.70)	< 0.001^*^
MCHC (%)	32.67 ± 1.14 (30.10–35.70)	30.13 ± 1.93 (24.90–35.70)	< 0.001^*^
RDW (%)	15.88 ± 1.13 (13.50–22.00)	20.48 ± 3.23 (14.90–26.70)	< 0.001^*^

The data are shown as mean ± standard deviation

*Hb* Hemoglobin; *Hct* Hematocrit; *IDA* Iron deficiency anemia; *MCH* mean corpuscular hemoglobin; *MCHC* mean corpuscular hemoglobin concentration; *MCV* mean corpuscular volume; *RBC* red blood cell count; *RDW* red blood cell distribution width; *TT* Thalassemia trait

* Mann-Whitney U test *p-*value < 0.05

### Evaluation of existing discriminant formulas and indices

To objectively evaluate the performance of our proposed discriminant model and fairly compare it with existing discriminant formulas and indices including BI [8], EF [9], E&F [10], G&K [11], MI [12], RDWI [13], RI [14], S&L [15], SI [16], SF [17], SiF [18], KF1 [19] and KF2 [19], the same dataset was used for evaluation of each. Their cut-off, Ac, Sn, Sp, MCC, YI, and AUC were shown in Table 3.

**Table 3:** Performance comparisons of existing discriminant formulas and indices proposed for differentiation of iron deficiency anemia from thalassemia trait

Indices/ formulas	Cut-off	Ac (%)	Sn (%)	Sp (%)	MCC	YI	AUC
BI = RDW	15	36.02	19.18	97.50	0.19	0.17	0.71
EF = MCV – 10 × RBC	15	52.15	45.89	75.00	0.17	0.21	0.70
E&F = MCV - RBC – 5Hb - 6.4	0	67.42	60.95	92.50	0.44	0.54	0.91
G&K = MCV^2^×RDW/100Hb	72	84.95	82.88	92.50	0.66	0.75	0.93
MI = MCV/RBC	13	54.30	47.95	77.50	0.21	0.25	0.75
RDWI = MCV ×RDW/RBC	220	67.20	60.96	90.00	0.42	0.51	0.92
RI = RDW/RBC	3.3	87.63	86.30	92.50	0.70	0.79	0.98
S&L = MCV^2^×MCH/100	1530	74.19	92.47	7.50	−0.01	0.00	0.31
SI = MCV – RBC - 3Hb	27	53.76	45.21	85.00	0.26	0.302	0.80
SF = MCH/RBC	3.8	37.63	26.71	77.50	0.04	0.04	0.68
SiF = 1.5Hb – 0.05MCV	14	24.73	31.51	0.00	−0.56	−0.69	0.02
KF1 = RBC/Hct + 0.5RDW	8.2	70.97	64.38	95.00	0.49	0.59	0.93
KF2 = 5RDW/RBC	16.8	89.79	89.04	92.50	0.74	0.82	0.98

*Ac* Accuracy; *AUC* Area under receiver operating curve; *Hb* Hemoglobin; *Hct* Hematocrit; *IDA* Iron deficiency anemia; *MCC* Matthew’s correlation coefficient; *MCH* mean corpuscular hemoglobin; *MCV* mean corpuscular volume; *RBC* red blood cell count; *RDW* red blood cell distribution width; *Sn* Sensitivity; *Sp* Specificity; *TT* Thalassemia trait; *YI* Youden’s index

**Fig. 3:**
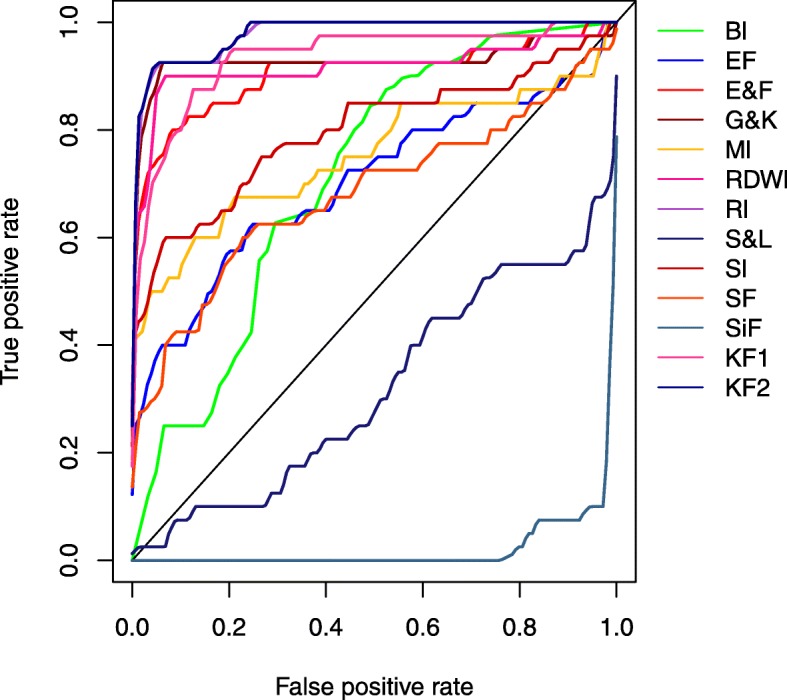
Performance comparison of existing discriminant formulas and indices using ROC curves

As noticed in Fig. 3, most existing formulas and indices achieved an AUC value that was greater than 0.5 [except for S&L (0. 31) and SiF (0.02)]. The highest MCC and AUC of 0.74 and 0.98, respectively, were achieved by the KF2 formula. Meanwhile, RI and G&K formulas performed well with the second and third highest MCC/AUC of 0.70/0.98 and 0.66/0.93, respectively. Meanwhile, the formulas and indices with the lowest values of MCC/AUC were SiF, S&L and EF (-05.6/0.02, 0.01/0. 31 and 0.17/0.70, respectively). These prediction performances indicated that only a few formulas and indices, e.g. G&K, RI and KF2, performed well on our dataset.

### Evaluation of the proposed discriminant model

**Fig. 4:**
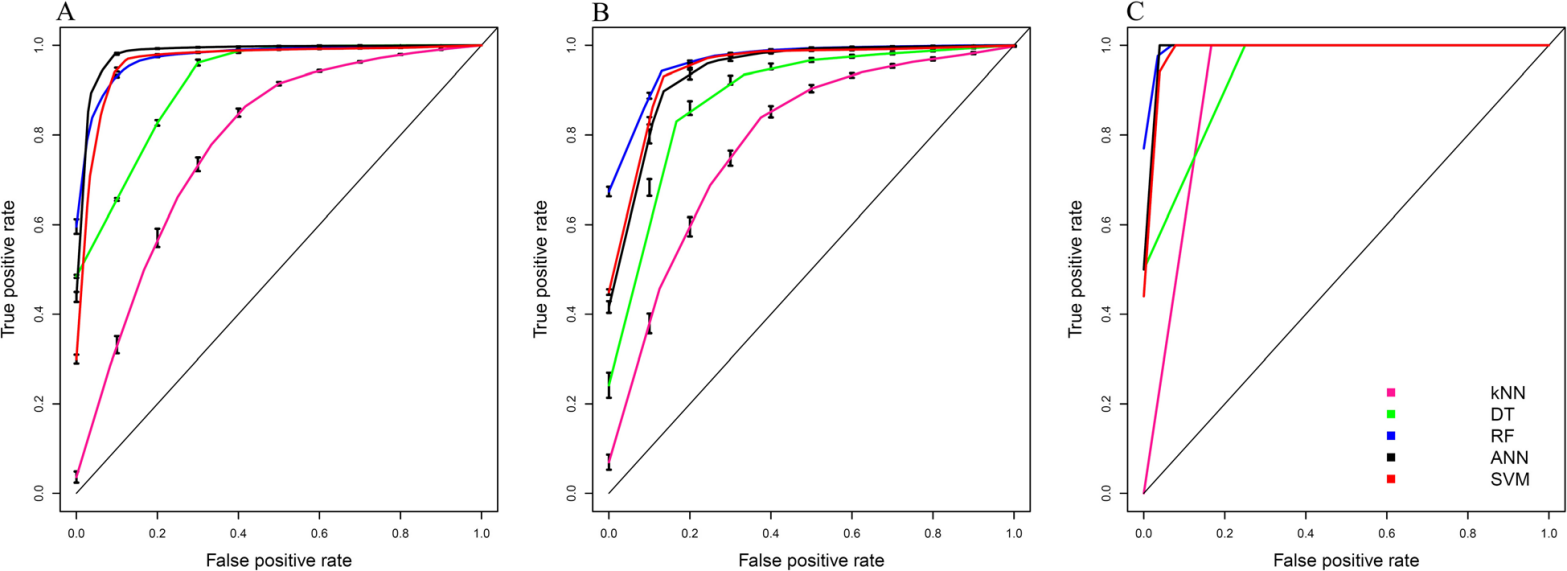
Performance comparisons among k-NN, DT, RF, ANN and SVM models using ROC curves over 5-fold CV (**a**) external test (**b**) and independent test (**c**), where k-NN, DT, RF, ANN and SVM models are represented by pink, green, red, black and blue, respectively

To develop a machine learning-based discriminating model, prediction performance depends on the parameter(s) used. In this study, five popular classifiers were considered, e.g. *k*-NN, DT, RF, ANN and SVM. To make fair comparison with existing formulas and indices, the same dataset was used. Furthermore, a 5-fold CV and external validation tests were carried out. As described above, the internal and external datasets were constructed with a random sampling procedure. To objectively evaluate the impact of the random sampling procedure, we repeated this construction 100 times. Table 4 and Fig. 4 list performance comparisons of various models using different methods over the 5-fold CV and external validation schemes.

**Table 4:** Performance comparisons between DT, RF and SVM models in differentiation of iron deficiency anemia from thalassemia trait

Classifier	Validation	Ac (%)	Sp (%)	Sn (%)	MCC	YI	AUC
*k*-NN	5-fold CV	92.36 ± 1.67	90.48 ± 3.62	92.80 ± 1.76	0.76 ± 0.06	0.83 ± 0.04	0.81 ± 0.07
	External	92.54 ± 4.26	90.09 ± 10.79	93.35 ± 4.22	0.77 ± 0.14	0.83 ± 0.13	0.80 ± 0.10
	Independent	90.20	83.33	100.00	0.82	0.83	0.85
DT	5-fold CV	98.03 ± 0.91	96.15 ± 3.40	98.54 ± 0.99	0.94 ± 0.026	0.93 ± 0.06	1.00 ± 0.00
	External	93.83 ± 4.10	86.86 ± 11.16	96.22 ± 3.77	0.82 ± 0.12	0.82 ± 0.04	0.92 ± 0.06
	Independent	92.16	86.21	100.00	0.85	0.85	1.00
RF	5-fold CV	94.17 ± 1.26	88.15 ± 3.49	95.75 ± 0.95	0.83 ± 0.04	0.83 ± 0.04	0.97 ± 0.01
	External	94.62 ± 3.29	90.07 ± 8.99	96.13 ± 3.19	0.84 ± 0.10	0.84 ± 0.07	0.98 ± 0.02
	Independent	92.16	86.21	100.00	0.85	0.85	1.00
ANN	5-fold CV	94.11 ± 1.31	86.75 ± 3.58	96.14 ± 1.06	0.83 ± 0.04	0.83 ± 0.04	0.97 ± 0.02
	External	93.78 ± 3.71	86.81 ± 10.84	96.22 ± 3.30	0.82 ± 0.11	0.83 ± 0.11	0.98 ± 0.02
	Independent	94.12	89.29	100.00	0.89	0.89	1.00
SVM	5-fold CV	95.05 ± 1.06	89.81 ± 2.66	96.45 ± 0.96	0.85 ± 0.03	0.85 ± 0.04	0.97 ± 0.01
	External	95.59 ± 2.76	92.49 ± 8.47	96.74 ± 2.59	0.87 ± 0.08	0.87 ± 0.10	0.98 ± 0.03
	Independent	96.08	92.59	100.00	0.92	0.92	1.00

The data are shown as mean ± standard deviation (100 times)

*Ac* Accuracy; *ANN* Artificial neural network; *AUC* Area under receiver operating curve; *DT* Decision tree; *k-NN* k-nearest neighbor; *MCC* Matthew’s correlation coefficient; *RF* Random forest; *Sn* Sensitivity; *Sp* Specificity; *SVM* Support vector machine; *YI* Youden’s index; *5-fold CV* 5-fold cross validation

Parameters of *k*-NN (*k*), RF (ntree, mtry), ANN (*size*, *decay*) and SVM (cost, γ) were optimized by a 5-fold CV procedure. Values of *k,* ntree, mtry, *size*, *decay,* cost and γ are 5, 200, 2, 4, 0.5, 8 and 0.5

As seen in Table 4, the DT model yielded the highest prediction results over the 5-fold CV with a mean Ac, Sp, Sn, MCC, YI, and AUC of 98.03, 96.15, 98.54%, 0.94, 0.93, and 1.00, respectively, while the SVM and RF models performed well with the second and third best prediction results. On the other hand, the SVM model was more effective than the DT model over the external validation test with a mean Ac, Sp, Sn, MCC, YI, and AUC of 95.59, 92. 49%, 96.74%, 0.87, 0.87, and 0.98, respectively. Interestingly, the SVM model still achieved levels of discrimination between IDA and TT on the independent dataset with means of Ac, Sp, Sn, MCC, YI, and AUC being 96.08, 92.59, 100.00%, 0.92, 0.92, and 1.00, respectively. As mentioned in the section *Data collection*, the dataset used in this study is imbalance among IDA and TT samples. Thus, we utilized a resampling approach to randomly generate a balanced internal set consisting of 32 IDA and 32 TT samples, the remaining samples consisting of 8 IDA and 114 TT sample were used as an external set. To objectively evaluate the impact of random sampling, we repeated this process with 100 independent iterations to generate the balanced internal and new external sets for constructing SVM models. The average prediction results (Ac and MCC) performed on the balanced internal and new external sets were (92.75%, 0.88) and (94. 30%, 0.77), respectively. These results indicated that SVM model can tackle the imbalanced dataset problem and provide desirable prediction results [48].

By observing the performance comparisons in Table 4 and Fig. 4, we conclude that DT model shows the highest performance level when evaluated by 5-fold CV, while SVM model outperform that other conventional models over the external validation test and independent dataset. This observation is consistent with the previous works [28, 49]. Furthermore, many studies have mentioned that the overfitting is the major problem of DT model [50, 51]. For convenience, this best predictor for discriminating between IDA and TT (based on SVM model) will be referred to as ‘ThalPred’.

For convenience, this best predictor for discriminating between IDA and TT (based on SVM model) will be referred to as ‘ThalPred’.

### Extracted important rules obtained from the RF model

The interpretable rules were established by using the RF model to demonstrate the combination of RBC indices for discriminating IDA from TT. Table 5 presents the eight interpretable rules as conditions in a simple linguistic manner, where *Frequency (%)* is the percentage of a data satisfying a condition, *Error* is the error percentage of a rule and *Prediction* is the outcome of a rule.

**Table 5:** The extracted interpretable rules derived from RF model in differentiation of iron deficiency anemia from thalassemia trait

Length	Frequency (%)	Error (%)	Condition	Prediction
1	63.98	0.00	Hb > 10.95	TT
2	15.05	0.00	RBC ≤ 4.5 and Hb ≤ 10.45	IDA
1	11.29	0.00	RDW ≤ 17.15	TT
3	4.84	0.00	RBC > 4.59 and Hb ≤ 10.95 and RDW > 17.7	IDA
4	2.15	0.00	RBC > 4.28 and MCHC ≤32.15 and RDW > 17.15 and RDW ≤ 17.7	TT
3	1.08	0.00	Hb ≤ 11.45 and MCHC > 31.35 and RDW > 17.4	IDA
1	1.61	33.00	Else	TT

*Hb* Hemoglobin; *IDA* Iron deficiency anemia; *MCHC* mean corpuscular hemoglobin concentration; *RBC* red blood cell count; *RDW* red blood cell distribution width; *TT* Thalassemia trait

The first rule, which covers 63.98% of the whole data, was constructed with single RBC indices, e.g. Hb. This rule has one criterion: if the value of Hb is larger than 10.95 g/dL, then the prediction is TT. The second rule, which covers 15.05% of whole data, was constructed with two RBC indices, e.g. RBC and Hb. This rule has two criteria: if (i) the value of RBC is equal to or less than 4.5 × 10^6^/μL and (ii) the value of Hb is equal to or less than 10.45 g/dL, then the prediction is IDA. The third rule, which covers 11.19% of the whole data, was depicted with single RBC indices, e.g. RDW. This rule has one criterion: if the value of RDW is equal or less than 17.15%, then the prediction is TT. The fourth rule, which covers 4.84% of the whole data, is constructed with three RBC indices, e.g. RBC, Hb and RDW. This rule has three criteria: if (i) the value of RBC is greater than 4.59 × 10^6^/μL and (ii) the value of Hb is equal to or less than 10.95 g/dL and (iii) the value of RDW is greater than 17.7%, then the prediction is IDA. The fifth rule, which covers 2.15% of whole data, was constructed with three RBC indices, e.g. RBC, MCHC and RDW. This rule has four criteria: if (i) the value of RBC is greater than 4.28 × 10^6^/μL and (ii) the value of MCHC is equal to or less than 32.15 g/dL and (iii) the value of RDW is greater than 17.15% and (iv) the value of RDW is greater than 17.7%, then the prediction is TT. The sixth rule, which covers 1.08% of whole data, was constructed with three RBC indices, e.g. Hb, MCHC and RDW. This rule has three criteria: if (i) the value of Hb is equal to or less than 11.45 g/dL, (ii) the value of MCHC is greater than 31. 35 g/dL, and (iii) the value of RDW is greater than 17.4%, then the prediction is IDA. The seventh rule covers 1. 61% of the whole data. If a query has RBC indices which do not satisfy any of the seven interpretable rules, then it is classified as TT with an error of 33.00%.

### Web-based tool implementation

**Fig. 5:**
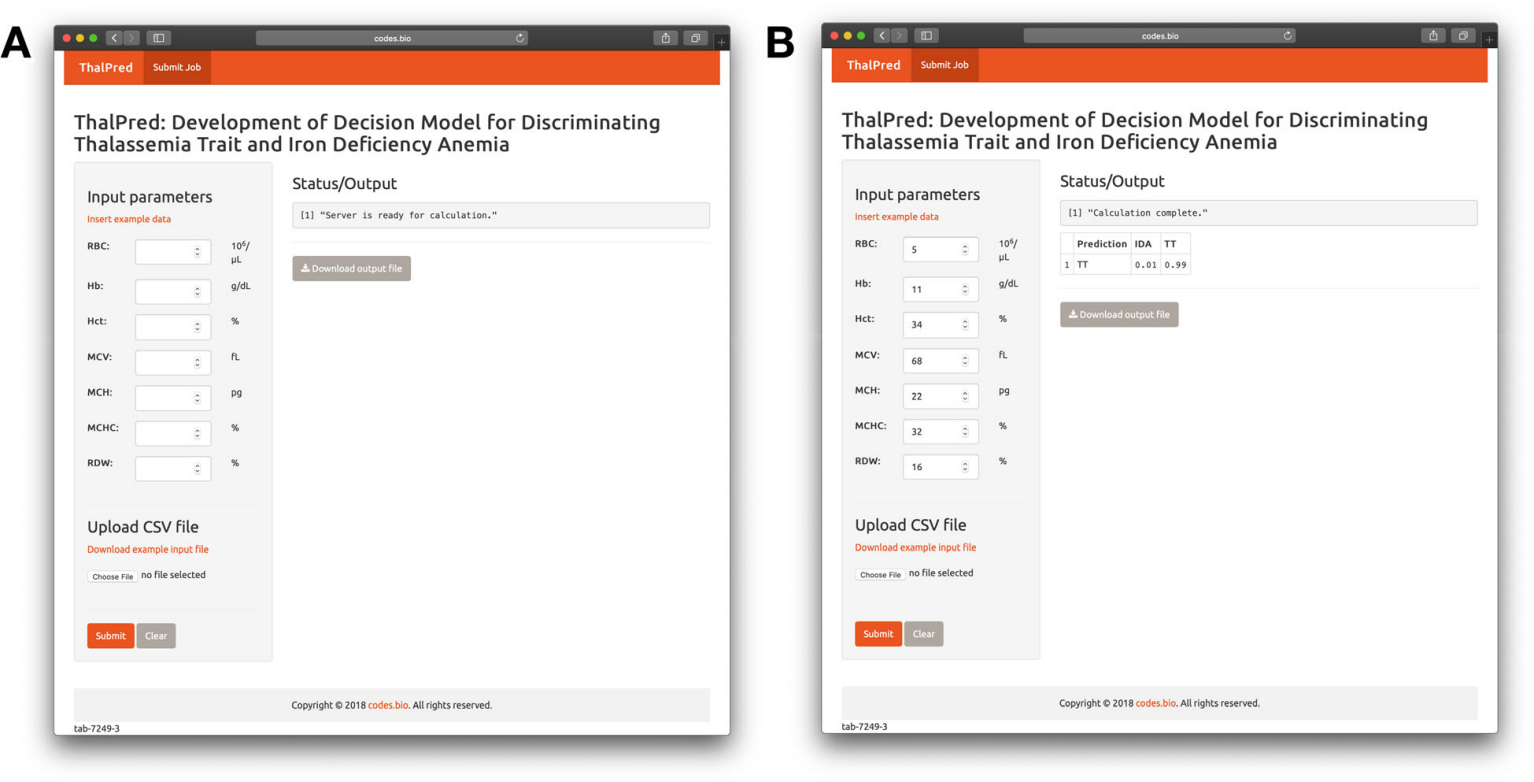
Screenshots of the ThalPred web-based tool before (**a**) and after (**b**, **c**) submission of laboratory data, which is available at http://codes.bio/thalpred/

For the convenience of health care team, based on the best model (e.g. SVM model) proposed in the present work, a publicly accessible web-based tool for ThalPred has been established. A screenshot of the ThalPred is shown in Fig. 5. Furthermore, to maximize user’s convenience, a step-by-step walkthrough of the procedures for using the ThalPred web based tool is provided below.

*Step 1.* Open the web-based tool at http://codes.bio/thalpred/.

*Step 2.* There are two options for prediction:

1) Key the single data in the *input parameters panel.*

2) Upload the query patient’s RBC indices (RBC, Hb, Hct, MCV, MCH, MCHC and RDW) into the input box by clicking on the *Choose file* button. The input data should be in CSV format. For example, of patient’s RBC indices in CSV format, click the *insert example data* above the input box*.* Finally, press on the *Submit* button to initiate the prediction process.

*Step 3.* Prediction results are automatically displayed in a grey box found below the Status/Output heading. Users can also download the prediction results as a CSV file by pressing on the *Download results* bottom.

## Discussion

Anemia is crucial problem occurring with population in Thailand and affecting to health and economic system. HMA, such as TT and IDA, are found commonly in clinical laboratory. Discrimination between TT and IDA group is essential for correct genetic counseling and effective treatment. RBC indices, consisting of RBC, Hb, Hct, MCV, MCH, MCHC and RDW, are generated from complete blood count (CBC) analysis using automated instruments. Therefore, using parameters from the CBC result for differentiation is of much interest. Generally, the RBC count, Hb, MCV, MCH and RDW are used to formulate a new index. As a result of the anemic condition, subjects with TT are found to have an increased RBC count [22, 52, 53]. While blood films from individuals with TT or IDA have shown different levels of anisocytosis (a condition of variation in sizes of RBC). Most of IDA patients appeared to have higher RDW than TT patients [22, 54]. The results of both univariate (Table 2) and multivariate (Fig. 2) analyses are agreeable with previous studies. Hence, one parameter is not enough to discriminate between these two conditions and all RBC parameters have potential role on the differentiation.

Previously, several discriminant formulas and indices have been proposed by different researchers for discrimination between IDA from TT [8–19]. However, these formulas and indices are not appropriate for every population. In Thailand, many researchers attempted to utilize these existing formulas and indices with different interest groups, e.g. school children [55], adults [56], anemic vegetarians [57], etc. In this study, the group of dataset composed of adults between 18 to 50 years old. Our finding showed that G&K, RI and KF2 performed well on our dataset. Interestingly, the top- three formulas and indices demonstrated the highest performance harboring of RDW and 2 of 3 consisting of RBC parameter.

Prior studies utilizing data from Thai people also found that most formulas containing RBC and RDW yielded a high performance. Pornprasert et al. suggested that SI and SF are the most reliable formulas [55], whereas RI was found to be most efficient in studies by Plengsuree et al. [56] and Sirachainan et al [18]. Conversely, S&L (which does not contain RBC and RDW) was proposed as a suitable index for differentiation in young Asians. It achieved 100% Sn and high Sp in Indian and Taiwanese [22, 54]. Our findings demonstrated consistent results to the previous publications that RBC and RDW play key roles in accurately distinguishing these two diseases. However, 89.8% Ac is not sufficient for medical decision making [58, 59]. Therefore, we attempted to increase the efficiency and accuracy of discrimination between TT and IDA in Thai adults by constructing a new model from hematological indices via machine learning approach.

As seen in Table 3 and Fig. 3**,** the prediction performances of the existing formulas and indices are still not satisfactory and there is room for improvement. Computational models based on machine learning approaches may further enhance prediction performance as well as provide effective large-scale analysis of available clinical data. The final prediction performances of the 5-fold CV and external validation tests were obtained by averaging the 100 corresponding performances of the internal and external sets, respectively. Furthermore, an independent dataset (Table 6) was used to assess the true predictive power of our proposed discriminating model.

**Table 6:** The prediction results derived from DT, RF and SVM models in differentiation of iron deficiency anemia (IDA) from thalassemia trait (TT)

No.	RBC	Hb	Hct	MCV	MCH	MCHC	RDW	Diagnosis	Prediction				
									*k-*NN	DT	RF	ANN	SVM
1	5.35	10.6	33.0	61.7	19.8	32.1	13.7	TT	TT	TT	TT	TT	TT
2	5.40	10.9	34.1	63.2	20.2	32.0	14.5	TT	TT	TT	TT	TT	TT
3	5.40	10.3	33.0	61.1	19.1	31.2	14.2	TT	TT	TT	TT	TT	TT
4	6.01	12.3	38.3	63.7	20.5	32.1	13.2	TT	TT	TT	TT	TT	TT
5	5.55	11.0	33.4	60.2	19.8	32.9	13.2	TT	TT	TT	TT	TT	TT
6	6.04	12.9	39.6	65.6	21.4	32.6	13.5	TT	TT	TT	TT	TT	TT
7	5.90	13.1	40.0	67.8	22.2	32.8	12.7	TT	TT	TT	TT	TT	TT
8	5.95	12.6	39.0	65.6	21.2	32.3	13.4	TT	TT	TT	TT	TT	TT
9	6.11	12.7	39.5	64.7	20.8	32.2	13.7	TT	TT	TT	TT	TT	TT
10	5.45	11.8	36.6	67.1	21.7	32.3	14.0	TT	TT	TT	TT	TT	TT
11	5.40	11.0	34.0	63.0	20.4	32.4	12.8	TT	TT	TT	TT	TT	TT
12	5.40	11.0	34.0	63.0	20.4	32.4	14.0	TT	TT	TT	TT	TT	TT
13	6.20	12.4	37.2	60.0	20.0	33.3	12.6	TT	TT	TT	TT	TT	TT
14	5.40	10.7	33.0	61.1	19.8	32.4	13.6	TT	TT	TT	TT	TT	TT
15	6.11	12.3	38.0	62.2	20.1	32.4	12.6	TT	TT	TT	TT	TT	TT
16	3.40	7.7	24.1	70.9	22.7	32.0	20.1	IDA	IDA	IDA	IDA	IDA	IDA
17	4.66	11.3	34.4	73.8	24.3	32.9	21.0	IDA	TT	TT	TT	IDA	TT
18	4.54	10.6	32.8	72.3	23.4	32.3	21.0	IDA	IDA	IDA	IDA	IDA	IDA
19	3.50	7.9	25.2	72.0	22.6	31.4	21.2	IDA	IDA	IDA	IDA	IDA	IDA
20	4.15	9.9	29.0	69.9	23.9	34.1	20.2	IDA	TT	IDA	IDA	IDA	IDA
21	3.90	8.9	28.0	71.8	22.8	31.8	20.2	IDA	IDA	IDA	IDA	IDA	IDA
22	4.17	9.9	29.0	69.5	23.7	34.1	21.1	IDA	IDA	IDA	IDA	IDA	IDA
23	3.85	8.5	27.5	71.4	22.1	30.9	21.3	IDA	IDA	IDA	IDA	IDA	IDA
24	4.24	9.8	30.5	71.9	23.1	32.1	20.2	IDA	IDA	IDA	IDA	IDA	IDA
25	4.83	11.1	34.0	70.4	23.0	32.7	19.0	IDA	TT	TT	TT	TT	TT
26	4.64	10.6	33.0	71.1	22.8	32.1	20.5	IDA	IDA	IDA	IDA	IDA	IDA
27	4.01	9.5	29.0	72.3	23.7	32.8	20.4	IDA	IDA	IDA	IDA	IDA	IDA
28	4.80	11.0	34.0	70.8	22.9	32.4	21.1	IDA	TT	TT	TT	IDA	IDA
29	3.65	8.4	26.0	71.2	23.0	32.3	20.4	IDA	IDA	IDA	IDA	IDA	IDA
30	4.00	9.2	28.0	70.0	23.0	32.9	20.1	IDA	IDA	IDA	IDA	IDA	IDA
31	4.45	10.2	31.4	70.6	22.9	32.5	19.8	IDA	IDA	IDA	IDA	IDA	IDA
32	4.44	10.3	32.0	72.1	23.2	32.2	21.3	IDA	IDA	IDA	IDA	IDA	IDA
33	4.56	10.4	32.0	70.2	22.8	32.5	19.4	IDA	IDA	IDA	IDA	IDA	IDA
34	4.84	11.0	34.0	70.3	22.7	32.4	20.7	IDA	TT	TT	TT	IDA	IDA
36	3.83	8.7	27.0	70.5	22.72	32.22	20.0	IDA	IDA	IDA	IDA	IDA	IDA
37	3.6	8.7	25.0	69.44	24.17	34.8	19.3	IDA	IDA	IDA	IDA	IDA	IDA
38	3.52	8.0	25.0	71.02	22.73	32	21.1	IDA	IDA	IDA	IDA	IDA	IDA
39	3.98	9.0	27.8	69.85	22.61	32.37	20.7	IDA	IDA	IDA	IDA	IDA	IDA
40	4.02	9.0	28.5	70.9	22.39	31.58	21.1	IDA	IDA	IDA	IDA	IDA	IDA
41	4.43	10.1	31.4	70.88	22.8	32.17	19.8	IDA	IDA	IDA	IDA	IDA	IDA
42	4.24	9.6	30.0	70.75	22.64	32.0	21.0	TT	TT	TT	TT	TT	TT
43	5.94	12.6	39.0	65.7	21.2	32.3	13.0	TT	IDA	TT	TT	IDA	TT
44	5.80	11.7	36.3	62.6	20.2	32.2	13.1	TT	IDA	TT	TT	IDA	TT
45	5.45	11.4	35.0	64.2	20.9	32.6	13.0	TT	IDA	TT	TT	IDA	TT
46	5.40	11.0	33.7	62.4	20.4	32.6	12.5	TT	IDA	TT	TT	IDA	TT
47	6.11	12.6	38.5	63.0	20.6	32.7	14.3	TT	IDA	TT	TT	IDA	TT
48	5.80	12.2	36.4	62.8	21.0	33.5	14.0	TT	IDA	TT	TT	IDA	TT
49	5.44	10.8	34.0	62.5	19.9	31.8	12.7	TT	IDA	TT	TT	IDA	TT
50	5.40	10.8	33.0	61.1	20.0	32.7	13.6	TT	TT	TT	TT	TT	TT
51	6.05	12.3	38.0	62.8	20.3	32.4	14.0	TT	TT	TT	TT	TT	TT

*ANN* Artificial neural network; *DT* Decision tree; *Hb* Hemoglobin; *Hct* Hematocrit; *IDA* Iron deficiency anemia; *k-NN k*-nearest neighbor; *MCH* mean corpuscular hemoglobin; *MCHC* mean corpuscular hemoglobin concentration; *MCV* mean corpuscular volume; *RBC* red blood cell count; *RDW* red blood cell distribution width; *RF* Random forest; *SVM* Support vector machine; *TT* Thalassemia trait. Parameters of *k*-NN (*k*), RF (ntree, mtry), ANN (*size*, *decay*) and SVM (cost, γ) were optimized by a 5-fold CV procedure. Values of *k,* ntree, mtry, *size*, *decay,* cost and γ are 5, 200, 2, 4, 0.5, 8 and 0.5

**Fig. 6:**
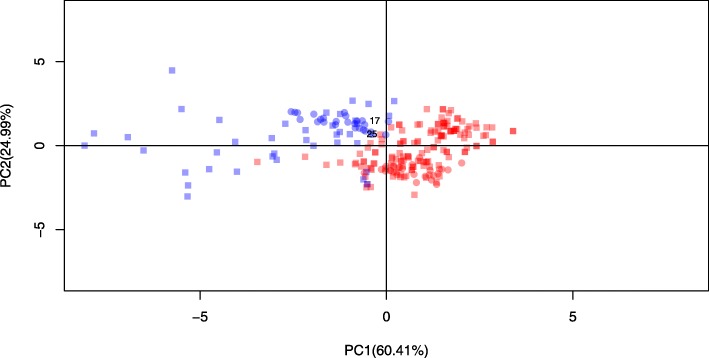
Multivariate analysis using principal component analysis (PCA) of internal (square) and external (circles) sets, where red and blue represent TT and IDA cases, respectively

As noticed in Table 6, the four or five prediction models give the prediction results on samples 17 and 25 as TT case, but the correct result is IDA case. These results might be due to the distribution of samples 17 and 25 is close to TT case, as illustrated in Fig. 6**.** Another reason supported our results is that thalassemia and IDA are most common etiologies of hypochromic microcytic anemia in Thailand therefore, both of them are close differential diagnosis and can coexist together. Prior studies showed that there is frequent occurrence of IDA in TT [60, 61]. Interestingly, this finding inspires us to perform future research for construction new efficient model to distinguish three groups including TT, IDA and coexisting of these two conditions.

As shown in Table 4, SVM and RF models perform well with the highest and second highest prediction results, respectively, for correctly discriminating IDA from TT. However, SVM models do not lend themselves as easily to interpretability. SVM is classified as black box model that could work perfectly with unknown data [62] because it overcame the problem of overfitting from DT [63]. According to DT model showed good performance on the training dataset but poor classification to other dataset in case the data is various detail and complex. RF could solve this limitation of DT [64]. Therefore, the seven interpretable rules (Table 5) extracting from RF were provided to represent criteria of each parameters for discrimination.

By observing the prediction results listed in Tables 3 and 4, we can clearly find that ThalPred (based on SVM model) has good discriminating power and outperforms all existing formulas and indices with the highest values of MCC (0.92), AUC (1.00) and YI (0.92). Improvements of MCC/YI with 18%/10 and 22%/12% for MCC and YI, respectively, were observed when compared with the best (KF2) and second-best (RI) existing formulas. Hence, ThalPred had better generalization capability for discrimination between IDA from TT than the existing formulas and indices. Further, ThalPred developed in the web server using SVM model [65] is stable and reliable to assist health care team for discrimination. This study is based on the small size [66, 67] of IDA and performed unbalancing dataset [68, 69]. So, the predictor may not be robust enough to apply on a very diverse dataset. As soon as more patients’ data require retraining as the new independent dataset to make the predictor more robust. This suggested that partition of the patients’ age for training dataset to make more sensitive and specific should be considered for the future development of effective models. The mobile application also be one choice for more convenient and available for all users.

## Conclusion

Discrimination between patients with IDA and TT is still a challenging problem due to the diversity of populations with anemia. Computational models can accelerate the process of screening HMA patients and save a lot of expenses and time. In this study, we have extracted an interpretable rule and established a web-based tool for discriminating IDA from TT. The prediction results for both cross-validation and independent validation tests on our laboratory data demonstrate the superiority of ThalPred over existing indices and formulas. Furthermore, a user-friendly web-based tool for ThalPred was established at http://codes.bio/thalpred/, by which users can easily obtain the prediction result without the need to follow the mathematical and computational details. We believe that the proposed ThalPred will supplement the existing indices and formulas as well as facilitate the health care provider.

## Data Availability

The datasets used and/or analyzed during the current study available from the corresponding author on reasonable request.

## Abbreviations

5-fold CV: Five-fold cross validation
Ac: Accuracy
ANN: Artificial neural network
AUC: Area under receiver operating characteristics curve
CART: classification and regression tree
CE: Capillary electrophoresis
DT: Decision tree
Hb: Hemoglobin
Hct: Hematocrit
HMA: Hypochromic microcytic anemia
IDA: Iron deficiency anemia
*k*-NN: *k*-nearest neighbor
LPLC: Low pressure liquid chromatography
MCC: Matthew correlation coefficient
MCH: Mean corpuscular hemoglobin
MCHC: Mean corpuscular hemoglobin concentration
MCV: Mean corpuscular volume
PCA: Principal component analysis
PCs: Principal components
RBC: Red blood cell
RDW: Red blood cell distribution width
RF: Random forest
SD: Standard deviation
Sn: Sensitivity
Sp: Specificity
SVM: Support vector machine
TT: Thalassemia trait
YI: Youden’s Index
