# Correction to: ThalPred: a web-based prediction tool for discriminating thalassemia trait and iron deficiency anemia

**DOI:** 10.1186/s12911-019-0977-7

**Published:** 2019-11-19

**Authors:** V. Laengsri, W. Shoombuatong, W. Adirojananon, C. Nantasenamat, V. Prachayasittikul, P. Nuchnoi

**Affiliations:** 1 Center for Research and Innovation, Faculty of Medical Technology, Mahidol University, Bangkok, Thailand; 2 Department of Clinical Microscopy, Faculty of Medical Technology, Mahidol University, Bangkok, Thailand; 3 Center of Data Mining and Medical Informatics, Faculty of Medical Technology, Mahidol University, Bangkok, Thailand; 4 Department of Clinical Microbiology and Applied Technology, Faculty of Medical Technology, Mahidol University, Bangkok, Thailand

**Correction to: BMC Med Inform Decis Mak (2019) 19:212**

**https://doi.org/10.1186/s12911-019-0929-2**

Following publication of the original article [1], the authors reported an error in one of the authors’ names. In this Correction the incorrect and correct author name are shown. The original publication of this article has been corrected.

Originally the author name was published as:

- C. Nantasenamart

The correct author name is:

- C. Nantasenamat
